# A Reflexive Thematic Analysis Exploring the Experiences of People Using the Curable App for Chronic Pain

**DOI:** 10.1155/prm/6996211

**Published:** 2025-11-25

**Authors:** Elisha Doi, Cormac Ryan, Niki Jones, Chris Penlington, Jagjit Mankelow

**Affiliations:** ^1^School of Health and Life Sciences, Teesside University, Middlesbrough, North Yorkshire, UK; ^2^Lived Experience Partner, School of Health and Life Sciences, Teesside University, Middlesbrough, North Yorkshire, UK; ^3^School of Dental Sciences, Newcastle University, Newcastle, UK

**Keywords:** chronic pain, digital health technology, mHealth app, qualitative research, reflexive thematic analysis

## Abstract

Pain management apps are gaining popularity for providing timely, accessible care. However, digital health intervention research is still in its infancy, with a limited understanding of how users experience and make meaning of these tools. This study explores users' experiences with the Curable app, considering its impact and the influence of contextual factors. Semi-structured interviews were conducted online with 12 Curable app users for chronic pain. Transcripts were analysed using reflexive thematic analysis through a critical realist lens. Five themes were conceptualised: (1) *The space between*, participants navigate a paradoxical space where trust coexists with scepticism and desperation with hope in a delicate balance. (2) *Tin Man and the Invisible Women,* follows the participants' journeys towards the Curable app, navigating systemic biases and cultural norms. (3) *It Takes a Village*, highlights the ‘village' of people, resources and modalities participants needed for effective pain care, beyond a solely biomedical approach. (4) *Enjoy Being in the Process of Becoming,* follows participants' interoceptive journey as they reconnect with the present moment, finding peace with their pain. (5) *Fiend to Friend*; *a story of neuroplasticity,* illustrates a shift in participants' fearful and combative relationship with pain towards a more conscious and compassionate companionship. The Curable app helped to fill gaps in existing care, illustrating the potential of digital tools when woven into broader ecosystems of support. However, as with all qualitative inquiry, these findings are situated and partial, reflecting the perspectives of well-educated, cisgender, English-speaking participants who chose to engage with this research.

## 1. Introduction

Chronic pain, experienced by approximately 30% of the global population, profoundly impacts an individual's physical, mental, social and spiritual well-being [1, 2]. The condition has been described as disrupting an individual's sense of self, fragmenting their identity into a previous ‘real' self and a constrained—sometimes unrecognisable—self [3]. There can be a sense of missing out on life, as pain can make it difficult to engage in activities that bring life meaning and joy [4]. This can leave individuals feeling disconnected from their values and society at large. Compounding the emotional and physical toll of chronic pain, many individuals describe their interactions with the healthcare system as a turbulent and often discouraging journey [3].

The lack of multidisciplinary pain clinics and long waiting lists makes timely access to effective care challenging, leaving many dissatisfied with the National Health Service (NHS) [5]. Despite the International Association for the Study of Pain (IASP) asserting pain management as a fundamental human right, 80% of the global population continues to lack access to this essential care [6, 7]. These prolonged delays exacerbate pain symptoms and deepen psychological distress, further deteriorating the individual's quality of life [8]. This underscores the urgent need for contextually responsive solutions to address these gaps in care.

With over three billion smartphone users worldwide, digital health technologies (DHTs), including mobile Health (mHealth) apps, have been explored as potential adjuncts to improve accessibility and timeliness in pain management services [9]. A UK-based survey of 2000 people found that 68% would support the integration of mHealth apps within the NHS, highlighting a public appetite for digital alternatives [10]. This growing interest is further reflected by the availability of over 500 pain management apps on the market [11].

Curable was identified as a commonly used app throughout the research teams' engagement with people living with chronic pain. The app was commercially developed with input from individuals with lived experience alongside a scientific advisory board of healthcare professionals (HCPs) and researchers. The app's artificial intelligence interface offers Curable users audio lessons on pain neuroscience, mindfulness exercises and journal prompts tailored by their feedback and preferences.

The research teams' patient and public involvement and engagement (PPIE) anecdotally highlighted a mixed reception, as some described Curable as helpful, while others found it less relevant or supportive. In recent reviews, Curable scored highly for quality, inclusion of psychologically informed content and self-management strategies [11–13]. Further to this, early trials suggest potential health-related benefits following Curable use [14, 15]. However, utility is shaped by the contexts and mechanisms through which users engage with an app and little qualitative work has explored this.

Therefore, this study aimed to explore the experiences of Curable users for chronic pain. It sought to understand the contextual barriers and facilitators to engaging with the app and how these influenced the perceived impact of Curable. Rather than focusing on whether Curable ‘works', the analysis explores how, when and for whom the Curable app was experienced as supportive or limiting.

## 2. Methodology

### 2.1. Study Design

This qualitative research was underpinned by a critical realist framework, which posits that an objective reality exists, though experiences are rooted in unobservable events and mechanisms activated by specific contexts [16]. Furthermore, our understanding of this reality is subjective and influenced by social and historical factors [17]. Critical realism facilitates a deeper comprehension of the participants' experiences with the Curable app and a contextualised understanding of the various mechanisms at play. This research was informed by reporting guidelines for qualitative research [18].

### 2.2. Participants

Ethical approval was granted by Teesside University, School of Health and Life Sciences Research Ethics Committee (19450). We recruited participants using advertisements on social media and chronic pain support groups, using a combination of purposive and snowball sampling. All participants were provided with study information sheets via email and subsequently submitted electronically signed consent forms before participation. There were no incentives for participation. Participants were eligible if they had independently accessed and used the Curable app for chronic pain, either currently or in the past. The research team did not provide access to the app; participants had downloaded and engaged with it on their own initiative. Although participants were required to be English-speaking, we did not impose restrictions based on country of residence and included individuals from the United States (3), India (1), New Zealand (1), and the United Kingdom (7). Ten participants described their gender as female and two as male, all identifying as cisgender. For further demographic information, see Table 1.

We conducted data analysis alongside the interviewing process to enable an iterative evaluation of information power, guiding the cessation of participant recruitment [19]. Recruitment stopped after 12 interviews as the richness and relevance of data in alignment with the research aims were prioritised over volume. Interviews ranged from 40 to 125 min and averaged 73 min, with a combined total of 14 h and 34 min.

### 2.3. Data Generation

The interview guide was piloted with three people with lived experience of chronic pain and iteratively improved to ensure comprehensibility. During the semi-structured interviews, the first author (ED) adopted a flexible conversational style to allow participants to express themselves freely, leaving space for the emergence of topics significant to their lived experiences. All interviews were conducted via Microsoft Teams, which uses industry-standard encryption to secure data [20]. Each interview was audio- and video-recorded, and a live transcription function was enabled with participant consent. Each transcript was manually reviewed for any apparent mistranslations, noting any nonverbal communication gestures observed on the recordings. Following this, we shared the reviewed transcripts with each participant to correct any further mistranslations and highlight any portions of the interview that they would prefer not to be directly quoted. Transcripts were then anonymised by assigning pseudonyms to each participant to protect their confidentiality. The anonymised transcripts were uploaded into NVivo to aid the analysis process.

We analysed the transcripts using reflexive thematic analysis, with an inductive orientation grounded in the data [21]. Reflexive thematic analysis involves the conceptualisation of meaning patterns across the participants' transcripts to form themes. The orientation of this research reflects a qualitative paradigm, viewing knowledge as partial and situated, often known as ‘Big Q' [18]. It stands in contrast to ‘small q' approaches, typically underpinned by positivist research values, by emphasising reflexivity and interpretation over generalisability. The analysis is positioned as a co-construction of meaning between the researcher, participants and the broader context.

The first author (ED) began by re-reading each transcript in NVivo, noting anything that felt interesting or relevant to the research aims. This stage was repeated using paper copies of each transcript to develop a deeper familiarity with the data. We refined early analytical notes into 69 semantic and latent initial codes, going through each paper and digital transcript concurrently. Each code was reviewed against its corresponding quotes to check its relevance to the research aims. During this review, codes were refined, merged or subsumed, leaving our total of 28 codes.

The first author (ED) continued with a digital and paper-based approach while developing preliminary themes, printing and cutting the codes out to facilitate a more intimate engagement with the data. Multiple iterations of grouping codes followed, as we considered underlying structures and tested them against the data to ensure they were grounded in the participants' accounts. This iterative process led to the conceptualisation of our five final themes. At this stage, the first author (ED) summarised each theme to encapsulate its central organising concept.

### 2.4. Positionality and Reflexivity

The philosophical underpinning and methodology of this research embrace the active role of the researcher in the analysis, acknowledging that themes materialise through the researchers' interpretative practice [22]. In becoming a ‘knowing' PhD researcher, ED kept a reflexive journal throughout to critically acknowledge and own her perspectives, recognising how her beliefs, intersectionality and experiences shaped the research. Working as a physiotherapist in chronic pain, ED (who led on data generation and analysis) brings an understanding of its complexities, impacting her ability to build rapport with participants. However, this professional background introduces a power dynamic, influencing how and what the participants felt comfortable sharing. ED is an able-bodied female, not directly experiencing the impact of chronic pain which positioned her as an ‘outsider' among participants. The remaining authors influenced the theme construction through discussions with ED on the transcripts, codes and themes in an iterative process. Niki Jones is a person with lived experience of chronic pain who has used the Curable app for her own pain management. Jagjit Mankelow, Cormac Ryan and Chris Penlington are HCPs, educators and researchers in the field of pain management with no personal or professional experience of using the Curable app.

## 3. Analysis

Through an interpretative and analytical co-construction of meaning, five themes were conceptualised: *(1) The Space Between, (2) Tin Man and the Invisible Women, (3) It Takes a Village, (4) Enjoy Being in the Process of Becoming* and *(5) Fiend to Friend: a story of neuroplasticity*. Each theme is discussed in depth, supported by quotes from the interviews, offering insight into the contexts that led participants to the Curable app and those influencing their engagement and perceived impact.

### 3.1. The Space Between

The Space Between captures the paradoxical nature of participants' experiences with the Curable app. This theme demonstrates how dualistic thinking and binary categorisation fail to capture the complexity of the human experience. It holds space for the existence of seemingly contrasting concepts, embracing the tangled nuance and complexity of the participants' journey to and with the Curable app.

Before discovering Curable, participants had placed their trust in the healthcare system, only to find themselves trapped in a vortex of hopelessness, where HCPs were perceived as gatekeepers of the belief in recovery. ‘*So I didn't see a doctor for a while after that. Felt like he was either saying I was making it up or there just wasn't hope'—*(Rosa). *‘In pain management, they'll never tell you that all these things can make the pain go away… like they're [healthcare professionals] not allowed to say that [recovery is possible]'—*(Karen). With an undertone of hopelessness and a sense of having exhausted all other treatment options, desperation was often cited as a motivator for trying the Curable app. Participants spoke of the devastating impact pain had on their lives, to the point where their existence felt so diminished that many believed they had ‘nothing to lose' by giving the app a chance. ‘*So when I used it, it helped me get through the day, helped me survive. Because that's the only thing I had… So it was complete and utter. Yeah, desperation for any kind of positive input or messages that there was hope'*—(Karen).

Amidst the desperation, the Curable app seemed to go beyond what the healthcare system was able to provide, offering participants a crucial lifeline: hope. ‘*[Curable] was the little ray of sunshine behind the bloody thunderclouds. That was a ray of hope… They're realistic when they're talking and sharing their information, and people are sharing their stories. It doesn't happen overnight. It is a freaking rollercoaster. It's a journey andthat isclear on there*'—(Gina). Optimism and hope were found in the Curable apps' realistic and relatable success stories, illuminating the possibility of recovery, a concept clouded by years of suffering and failed attempts at relief. However, this hope was accompanied by scepticism, a tension triggered by the app's name. Perceived as a marketing ploy, those who viewed chronic pain as incurable felt the *Curable* name was overpromising an unattainable outcome. ‘*[The name was] part of the resistance to using it to start with, because… I'd learned it wasn't curable.…it's a constant state of recovery'—*(Jake)

While scepticism existed around the Curable app, participants' positive experiences with mind-body practices and health apps lent it credibility. ‘*I'd known that [another app] had been useful. So I suppose I had [belief] that an app could be useful'*—(Sharon). The relatable recovery stories and integration of lived experiences in the app fostered trust, contrasting a distrust felt towards HCPs. ‘*I wouldn't have tried the app if my doctor recommended it… [but] there's plenty of lived experience perspective in [the Curable app] as well, which I think is key because like I spoke to you about before, that's where the trust lies*'—(Jake). Despite this distrust in HCPs, participants found a paradoxical sense of reassurance in their involvement in the app's curation, reflecting lingering beliefs in biomedical authority and the idea that ‘doctor knows best'.

The timing of participants' discovery and engagement with the Curable app introduced another duality. Familiarity with the app's underpinning research and theories was both validating and limiting, providing a foundation for belief in its utility while simultaneously dulling its impact. ‘*I think because I'd already learnt a lot of it. It wasn't as helpful as it could have been'*—(Trudy). Similarly, the cost of the app served as both a deterrent and a motivator. On one hand, participants expressed their financial difficulties and hesitation after having already invested significantly in seeking pain relief. Yet, on the other hand, the financial investment encouraged them to maximise their engagement with Curable: ‘*I'm a Yorkshireman. I've got short arms, long pockets, you know, I want my money worth'—*(Jake).

This theme highlights participants' dance in the space between dualities as they navigate their experience with the Curable app, embodying Carl Jung's insight, ‘Only the paradox comes anywhere near to comprehending the fullness of life' [23]. Their experiences resist a simplistic narrative, revealing a complex interplay between desperation and hope, trust and scepticism. The Space Between encapsulates this paradoxical space where years of unmet expectations shape a delicate balance of optimism and caution.

### 3.2. Tin Man and the Invisible Women

Tin Man and the Invisible Women explores the impact of gender constructs on the participants' experiences with chronic pain, synonymous with the heartless Tin Man in The Wizard of Oz [24] and the work of Caroline Criado Perez [25]; this theme follows the journey participants of each gender described taking towards the Curable app, navigating societal expectations and systemic gender biases.

The historical overrepresentation of men in research has led to a healthcare system primarily designed for them, driving systemic gender inequalities [26, 27]. This bias was deeply felt by female participants, as their symptoms were repeatedly attributed to life stages and trivialised, rather than taken as legitimate health concerns. ‘*No one listened to me. They didn't believe me. That's your periods [they would say]'—*(Gina). Female participants found their pain quietly judged as malingering or dismissed as solely psychological, reflecting the outdated hysteria discourse rooted in andronormativity. Echoing this, experimental research shows that women are disproportionately referred to psychological support, a decision seemingly rooted more in gendered bias than in clinical judgement [28, 29]. While this highlights the role of gender bias in shaping referral pathways, it is important to recognise that psychological support can be beneficial for chronic pain and is encouraged in management guidelines [30]. However, after having already faced significant invalidation, such a referral can be viewed as further dismissing their pain. ‘*I thought [the healthcare professional] was telling me it was in my head'*—(Sharon). This appeared to foster a reluctance among some female participants to consider a psychological intervention, estranging them from a key dimension of pain management.

Wary of further stigma and invalidation, female participants began to self-silence and avoid care altogether, creating a sense of resignation. ‘S*he didn't realise how much pain I was in… I wasn't going to say I'm suicidal because they might section me*'—(Karen). The participant's hesitancy to express the full extent of her pain highlights a fear of being met with a punitive or dismissive response, illustrating the alienation faced. This quiet endurance illuminates the healthcare system's failure to truly ‘see' these women, echoing their feeling of invisibility, both in medicine and in society at large. Through validation, Curable provided a contrast to traditional healthcare, offering a space where participants' pain was acknowledged rather than dismissed. ‘*But also knowing [from the Curable app] that they were real conditions… [having] the validation that it doesn't mean that there isn't pain'—*(Amy). Feeling seen, the female participants appeared more open to engaging with psychological interventions as part of a holistic approach.

While women bear the burden of systemic gender bias, the male participants described the constraint of hegemonic masculinity. The Cure [31] proclaims, ‘Boys Don't Cry', reflecting the pressure on males to ‘man up' since cultural ideals equate masculinity with emotional suppression [27]. Jake recalled feeling this pressure on the day of his father's funeral: ‘*Come on, you're the man of the house now… that put a lot of pressure on me as a young boy. I mustn't show weakness now because I'm the man of the house*'. This internalised pressure to suffer in silence mirrors the healthcare system's tendency to medicalise men's pain with pharmaceuticals, disregarding its psychological dimensions [29]. In denying men access to holistic coping strategies, this cycle of repression can contribute to broader societal issues, including higher rates of substance abuse and imprisonment [32, 33]. ‘*I started smoking a lot because it gave me a little relief with my pain'*—(Terry).

Conforming to these patriarchal ideals can trap men in isolating patterns of endurance, which can give rise to the darkest of thoughts—reflected in the stark reality that 75% of suicides are male [34]. ‘*It reached a point where I had suicidal thoughts'*—(Terry). Breaking free from these deeply ingrained gender constructs can be challenging. Jake remarked his ‘*lightbulb moments'* often came from men as he reflected on a podcast featuring two male doctors; ‘*Emotional traumas can create physical pain, and when I heard the doctor say that line. It was like that's me'.* While the podcast is unaffiliated with the app, one of the doctors is on the Curable advisory board. ‘*And it really then encouraged me to start pursuing the psychological side of things… that's been part of the recovery process is [Curable] helping me let go of a lot of the anger'—*(Jake). It was as if this validation from a male figure permitted him to begin exploring his repressed emotions. The Curable app appeared to offer the male participants the opportunity to consider the psychological dimensions of their pain, alongside other drivers, without the fear of societal scrutiny.

Michel Foucault's [35] conceptualisation of power and discourse intersects with Dana Jack's [36] concept of self-silencing, shedding light on how gender constructs shape not only societal expectations and healthcare practices but participants' emotional and behavioural responses too. This theme illustrates how participants suppressed their voices and needs, perpetuating cycles of invisibility and alienation in chronic pain experiences. For many, this drove them to seek alternative solutions, with the Curable app offering a powerful contrast to traditional healthcare. Curable offered participants a safe space, filled with messages of validation and hope, where they finally felt seen and understood.

### 3.3. It Takes a Village

Echoing the African proverb, ‘It takes a village to raise a child', this theme highlights the ‘village' of people, resources and modalities that participants felt were essential alongside the Curable app. Participants valued a holistic and collaborative approach, reflecting the multifaceted nature of chronic pain and the inadequacies of treating pain solely through the biomedical lens. This theme illustrates the participants' need for a supportive, integrative community of HCPs, peers and loved ones in addressing all the layers of the individual's unique pain experience.

As chronic pain does not conform to the dominant biomedical model, which seeks tangible evidence of pathology, it is often met with disbelief and judgement by HCPs. ‘*Part of the struggle and the mental pain, hard for me at that time, was I wasn't being believed from the doctors'*—(Gina). The persistent dismissal and disbelief they faced reinforced a need to assert the legitimacy of their pain, intertwining their chronic pain with their sense of identity; ‘*so many people have been told for so long it's in your head and been invalidated. I think it's really easy to hold on to your diagnosis because it's real and it means that you weren't making it up'*—(Amy). This may contribute to participants' initial hesitation to explore psychological factors behind their pain, as doing so could challenge the notion that their pain was structural and therefore ‘real'.

There was a sense of abandonment that extended beyond medical settings, as both HCPs and loved ones struggled to understand or acknowledge their suffering; ‘*Going to the doctors, going to see everybody. No one listening, no one understanding*'—(Gina). The stigma surrounding these invisible conditions left participants feeling ostracised from society, suffering in silence. Participants described their world becoming smaller as they withdrew from social activities where their pain was overlooked or misunderstood. Stigma and isolation compound their suffering, a pattern associated with increased pain, disability and distress [37]. ‘*You know, being in pain is really hard. But there's no comparison to the loneliness you feel of not being seen'—*(Karen). This sense of invisibility reveals the isolating impact of the societally entrenched biomedical understanding of pain, reflecting the widespread marginalisation experienced by those living with chronic pain.

When participants' experiences were marked by alienation and marginalisation, the need for community and belonging was especially significant. ‘*I knew that my needs weren't getting met on a very basic level of connection, and that's where you have to start from'—*(Karen). The collective experience of suffering shared within the Curable community appeared to offer a sense of solidarity as participants no longer felt like outsiders. In this virtual ‘village' of allies, their struggles were recognised and validated, dismantling the isolating stigma and the need to constantly prove the legitimacy of their suffering. ‘*It was almost like the app put an arm around you'—*(Jake). For many, this sense of community and belonging was a foundation upon which they could begin to rebuild their sense of self and their belief in the possibility of recovery.

Although the Curable app played a pivotal role in many of the participants' journeys, it was acknowledged as a complementary tool rather than a standalone solution; ‘*I wouldn't say that's [Curable app] the only thing that's transformed things*'—(Jocelyn). Some used the app alongside other forms of care, such as physiotherapy, psychology or a structured pain management programme, and the alignment between both reinforced their trust in the approach. Others used the app independently of the healthcare system, though it remained part of a broader approach, supplemented by resources, such as books, podcasts and other online education. ‘*When I say Curable, it's not the only thing I used. So it's that approach around it…what they call the mind-body approach. So whenever I say Curable,* that is *what I mean because I used quite a lot of things*'—(Terry). This blended approach allowed participants to tailor their management to their own unique and individual needs, using the app as one piece of a larger integrative puzzle.

The participant journeys came full circle, as after finding relief through the app and other resources, many felt a deep desire to pay it forward and help others navigate the challenging currents of chronic pain. ‘*There's something about feeling that you were contributing to possibly other people feeling less in pain or less alone'—*(Amy). For some, this meant engaging with research to spread hopeful messages of recovery, while others were retraining to become pain coaches; ‘*you know, doing what I do with the health coaching and the engagement with the pain classes is recovery in itself for me'—*(Jake). This collective spirit of giving back underscores the theme ‘it takes a village'—not only to find healing but to sustain it and help others along the way.

### 3.4. Enjoy Being in the Process of Becoming

This theme explores participants' interoceptive journey with the Curable app, alongside other resources and support. It illustrates an energy shift from the fruitless search for pain resolution to reclaiming their lives with purpose and joy. Participants reconnect with their inner being and the present moment, finding peace with their pain as they embrace the journey of becoming.

An undertone of intensity ran throughout the participants' narratives, revealing high-achieving tendencies and exceptionally high self-expectations. This was apparent in the challenging juggle of multiple roles, trying to be everything to everyone; ‘*I was trying to care for him [unwell partner] and work and care for the children…So, you know, ended up being a sh*^*∗*^*t employee, a sh*^*∗*^*t parent and a sh*^*∗*^*t carer whilst trying my hardest at everything'—*(Jocelyn). Amidst this constant balancing act and dutiful self-sacrifice, little space was left to prioritise their own health and well-being. Participants often adopted a ‘mind over matter' mentality, pushing through the pain as their critical inner voice seemed to perpetuate feelings of inadequacy and unworthiness. The pressured expectation to succeed despite pain appeared to create a fragmented sense of self, entangling their self-worth with productivity and achievements. In anticipation of a flare-up hindering productivity, participants described a pre-emptive drive to stay ahead; ‘*the migraines themselves really pushed me to perfectionism…I never know when I'm gonna get a migraine, and therefore I have to get everything done early'*—(Debbie). Perfectionism seemed to become a defence mechanism against the unpredictability of pain, overcompensating to gain a sense of control.

The intensity with which some participants approached the Curable app reveals a potential drawback, particularly for individuals with high-achieving or perfectionist tendencies. Trudy shared ‘*I did the journalling, which I think would have been helpful but… I just think I did too much of it. I mean hours and hours of it, day after day… it upped my pain, and it lasted for a long time'.* This participant's experience demonstrates how engaging with a therapeutic practice too intensely can be counterproductive and amplify pain for some. Rosa echoed this sense of urgency; ‘*I treated Curable like crack. Like it was, I got to know everything about this. And so I just whipped through the lessons really, really fast'.*

Many were consumed by future-oriented thinking, whether striving to be cured or working excessively to stay ahead of anticipated pain, disconnecting them from the present moment. Tolle [38] suggests that this state of waiting—wanting to be in the future, rather than the present—creates inner conflict with the here and now. An intense focus on avoiding pain can amplify the very suffering one is trying to escape [39], particularly when pain is positioned as a problem to be solved rather than an experience to be understood. Curable encourages users to let go of the intense quest for pain resolution and to approach the present moment with curiosity and safety, rather than fear and urgency.

The Curable app appeared to help participants recognise the importance of grounding themselves in their present reality as they realised that acceptance, rather than resistance, is the foundation upon which to build*. ‘If I'm constantly fighting where I am, then I'm not starting from where I am… There's no point starting from anywhere other than acceptance. I think that's probably been the sort of, the most profound part of it'—*(Amy). This reflects the principles of mindfulness which encourage a non-judgemental awareness of internal and external experiences [40]. Shifting away from the urgent drive to resolve pain or the relentless need to prove worth through productivity seemed to foster equanimity in participants—a state of balanced, non-reactive awareness of their symptoms and life situation. ‘*I expected that if I got better, I'd become more productive. I was already doing the work of five people. I thought I'd do the work of 10 and after getting better, I decided I didn't want to be more productive. I just wanted to be happier… It sounds so cliche, you know, stopping to smell the roses kind of thing'*—(Debbie). With acceptance of the present moment, comes a sense of peace and stillness [38].

Building this acceptance seemed to lay the groundwork for a deeper self-awareness, as participants became the observers of their unhelpful patterns and conditioning. Jocelyn reflected on the insights gained from the journaling activity; ‘*I realised I've been telling myself that I was a sort of happy, reasonably content person, but actually writing down and articulating my moods showed that I had a lot of stress and upset in my everyday life. So that was quite revelatory to me*'. The Curable app appeared to broaden participants' attention from the physical manifestations of their pain to illuminate the previously overlooked cognitive, psychological and social dimensions. ‘*This was all stuff from the Curable app, like identifying perfectionism and how I would speak to myself when I would start feeling a headache coming on, that it was a failure'—*(Kylie).

This growing self-awareness appeared to naturally pave the way for participants to slow down and cultivate a more compassionate relationship with themselves, moving from self-criticism to self-nurture. ‘*[Curable] taught me to be a little bit more selfish… my care went elsewhere and [I] didn't care for me. But now I do'—*(Jake). This gentle approach created a sense of calm as participants learnt the skills to regulate their nervous system; ‘*I do think that understanding and learning more has helped me not be so in fight and flight all the time' *(Gina). The effects of this calm extended beyond their internal experience by reducing interpersonal tension and creating a more peaceful, supportive environment both within and around them. ‘*I feel like I have so much more space to respond rather than react… there's just a lot more space to be able to hear what somebody's saying and not just immediately be defensive or triggered'—*(Rosa). With acceptance and self-awareness intertwined with self-compassion, participants seemed to move beyond seeking mere pain relief towards a deeper sense of peace and presence.

Participants expressed an unexpected feeling of gratitude for their pain, as it led them to embrace a healthier lifestyle and a deeper understanding of self. As Amy reflected, ‘*it's probably worth the things that I've lost, in order to get the things that I've gained'.* This powerful sentiment highlights how hardship can lead to personal growth and well-being that might have otherwise remained unexplored. There was a sense of deeper fulfilment as the Curable app appeared to redirect participants' focus from the elusive cure to quality of life; ‘*If you're searching for a cure, then your pain will persist… I don't aspire to be pain free. You know? I just aspire to try and live a better life'—*(Jake). Participants seemed to regain a sense of self beyond suffering and described finding joy as they reconnected with their values, cultivating a more meaningful way of being. ‘*[I'm] now just approaching life more playfully and more joyfully, more gratefully… [Curable] just had this general, you know, glow that spread to everything'—*(Debbie).

By reconnecting with a state of being, rather than an endless stream of doing, participants learnt to be present with their experience and redefine their identities beyond pain and productivity. As the participants embraced this mindful presence over perfection, they made peace with the space between who they are and who they want to be. This appeared to enable participants a greater capacity for joy, empowering them to live fully with purpose.

### 3.5. Fiend to Friend: A Story of Neuroplasticity

This theme represents a shift in the perception of pain from an uncontrollable fiend to be fought, to a protective friend providing guidance. Learning about the multifactorial nature of pain while filling their toolbox with strategies, participants seemed to move away from a fearful and combative relationship with their pain towards a more conscious and compassionate companionship.

Before finding the Curable app, participants widely believed that pain resulted from structural damage, a concept deeply ingrained in society from the biomedical model of health [41]. This perspective fosters a reliance on HCPs to ‘fix' the structure and thus eliminate the pain, reflecting an external locus of control. By placing their recovery in the hands of the healthcare system, participants felt powerless in their suffering; ‘*you've got some damage, you go to the doctor or the hospital and get them to fix whatever'—*(Gina). Structural explanations for pain further ingrain this biomedical thinking, creating a sense of fragility about the perceived damaged tissue. ‘*[I was told] we can see compressions at L4/L5… The way that MRI was explained to me. I came off the phone [with the GP] and I literally said to my partner I'm gonna be in a wheelchair in 10 years…how do you function with the spine that's falling to pieces*'—(Jake). This quote illustrates how scan findings and structural explanations—when presented without appropriate context—can heighten fear and despair, triggering catastrophic thinking about seemingly inevitable deterioration.

When pain persisted despite ‘fixing' the structure, some internalised self-blame, believing their continued suffering was a personal failure. ‘*So I spent quite a lot of years blaming myself for the fact that I was in pain because [I thought] I wasn't trying hard enough to get rid of it'—*(Amy). Others continued their search for the ‘right' doctor or treatment; ‘*So I kept changing doctors. Just trying to, going to the most like expensive. I thought that was what will solve my issue right? Like the best doctor'—*(Terry). Both responses appeared to intensify participants' feelings of fear and hopelessness, aligning with the concept that a damage-focused approach to chronic pain reinforces a cycle of ineffective problem-solving that exacerbates distress and suffering [42]. This sense of having no control and no resolution pushed some towards extreme thoughts, echoed by the haunting sentiment, ‘*Well, if it ever gets really bad, I'll just kill myself'—*(Debbie).

The Curable app, alongside other resources, appeared to facilitate a shift in participants' understanding as they came to view pain as an experience created by the brain; ‘*I [now] understand pain as an opinion of the brain and not evidence of harm or injury'—*(Rosa). Moving away from the structural perspective rooted in the biomedical model, participants began to recognise the influence of emotions on their pain experience; ‘*I think that was the biggest surprise to me… the emotional content and drivers of pain*'—(Jocelyn). This newfound understanding of pain's biopsychosocial nature appeared to foster a greater sense of agency among participants, as the Curable app introduced strategies to address the different dimensions. With this shift towards an internal locus of control—where participants had the power to influence their pain—fear and urgency seemed to subside. ‘*It is a sensation that can be turned down just like a volume knob. And I know how to do it [now] and I think that's a big part of the recovery is I feel like I'm in control now'—*(Rosa).

Reflecting on the bidirectional relationship between pain and fear, Jake echoed this transition toward self-regulation; ‘*If you don't fear pain, it doesn't stick around as much ‘cause it has less to thrive on. It's almost like a predator. It predates on fear. It predates on anger. It predates on those emotions'.* In a power shift, Jake became aware of his repressed emotions fuelling his pain persistence, realising the possibility of change. By relinquishing the exhausting cycle of resistance and avoidance, Jake began approaching pain with curiosity and openness; ‘*I now see pain as my friend. I don't see it as my enemy. I don't see it as something I've got to fight or battle or rage against. If I'm experiencing pain, I sit with it and I explore emotions'.* Rather than a foe to fight, pain was reframed as a messenger and an experience to engage with rather than resist.

The concept of neuroplasticity played a role in creating this new narrative for pain; ‘*I think understanding neuroplasticity has been just amazing… the brain and the body together are trying to look after you and some signals get messed up and sometimes the signals for danger are still going to try and protect you, even when the danger isn't there anymore'—*(Amy). This miscommunication revealed to participants how the brain can develop habitual pain pathways, resulting in an overly protective system. Understanding that the brain can become conditioned to send danger signals and trigger pain—even in the absence of injury—helped participants realise their pain was changeable. ‘*I think what was surprising was the habitual nature of pain. And I think I learned a lot about how these neural pathways are set up and how you can disrupt them essentially'*—(Jocelyn). A sense of hope emerged as participants learnt that the brain can be retrained to reduce these false alarms by ‘*getting your brain to believe that it's safe'—*(Amy).

As observed in other studies, this shift in participants' understanding of pain not only reduced their symptoms but also empowered them to reclaim agency over their lives [43, 44]. ‘*So by using the [Curable] app and understanding about that, I could decrease the level and intensity of my pain'—*(Gina). This empowered approach opened a path to recovery for some, or living well with pain for others, as they developed the knowledge and skills to influence their pain. ‘*It puts me where I am today, which is living well with pain, not rid of it…It was like having my own psychologist and my own health coach in my pocket whenever I wanted them. And it really took, for me, the recovery journey to another level'*—(Jake). *‘So even if there's no more recovery, and even if the pain doesn't completely go, I'm still, yeah, having a much better time than I was before I knew about pain science'*—(Sharon). *‘It's definitely life changing for me, I mean. The best thing physically that's happened to me ever, really. It's the most helpful medical thing I've ever*… *I mean, I'm practically cured'*—(Kylie).

## 4. Discussion

This research moves beyond the functional mechanics of the Curable app to the human experience of it. One that is both situated within and continually shaped by the complex social, relational and structural worlds in which participants live. The shifts that traverse the themes—from disconnection to belonging, silenced to validated, and resisting to accepting—capture the personal transformations facilitated by Curable, while also illuminating the systemic transformations required in how pain is understood and responded to.

At the heart of this study was a primal yearning from participants for their pain to be acknowledged and believed, not solely by HCPs but by society itself. While validation has been framed by some scholars as a prerequisite for engaging with digital pain interventions [45], this research presents a more complex narrative. The invalidation, dismissal and marginalisation encountered within healthcare appeared to steer participants towards Curable. In this context, Curable went beyond a tool for symptom management, offering a space where participants felt seen, heard and understood. Though this virtual validation was not unique to this research or the Curable app [46, 47], its presence here highlights both a gap and an opportunity in healthcare. We hope this research invites a deepening of professional practice, a capacity to truly witness and validate the pain experience. DHTs should not be seen as substitutes for relational care, but as an extension of it—carrying validation, continuity and compassion beyond the clinic walls.

Participants' healing and recovery from persistent pain is conceptualised within this research as a collective endeavour, supported by networks of care rather than individual effort alone. Contrary to concerns that DHTs may deepen isolation, Curable appeared to cultivate a sense of belonging and connection among participants, resonating with other qualitative literature [47–49]. Integrating digital tools like Curable into an ecosystem of support—encompassing HCPs, peers and loved ones—can help meet all the layers of a pain experience. This echoes the growing calls for blended approaches [45, 50–52]. Researchers, clinicians and policymakers must recognise the interdependence of formal healthcare, informal support and digital technologies in shaping compassionate and sustainable systems of care. One practical way to enact this is by weaving the expertise of people with lived experience throughout clinical practice, research and the design of DHTs.

Despite efforts to offer a non-judgemental and curious presence, participants were aware of the researcher's clinical background which may have influenced how openly they shared experiences of healthcare. However, this study's reflexive orientation and the interdisciplinary research team—combining physiotherapy, psychology and lived expertise—enhanced the analytical and theoretical depth of the work. This analysis must be understood within certain boundaries. Participants were well-educated, cisgender and English-speaking individuals who self-selected to engage with this research. Those who chose to participate may have been more likely to have had positive experiences with the app and pain recovery more broadly. These boundaries do not diminish the authenticity of the participants' experiences or the analytical interpretation but reflect the situated, co-constructed nature of qualitative knowledge.

## 5. Conclusion

By centring the voices of Curable users and situating their experiences within wider cultural and institutional contexts, this study not only offers insights into how digital tools like Curable can transform the relationship with chronic pain but also calls for a deeper societal reckoning with the way we view, treat and respond to pain. It invites future research to deepen attention to intersectional identities, systemic inequities and the co-construction of recovery across individual, social and systemic dimensions.

## Figures and Tables

**Table 1:** Demographic information.

Gender	
Female	10
Male	2
Age	
24–34	1
35–44	1
45–54	8
55–64	1
65+	1
Ethnicity	
White British	7
White American	3
Asian	1
Pakeha	1
Working status	
Working full time	3
Working part time	2
Unemployed	2
Self-employed	1
Long-term sick	1
Retired	3
Religion	
Christian	3
Hindu	1
Islam	1
Agnostic	7
Educational background	
College/vocational	4
Bachelor's degree	3
Master's degree	2
Doctorate	3
Primary pain diagnosis	
Fibromyalgia	2
Chronic migraines	4
Ehlers–Danlos syndrome	1
Chronic pain	5
Duration of pain	
2–6 years	5
10–20 years	3
30 years +	4
Level of social support	
Limited	3
Moderate	2
Strong	7

## Data Availability

The data that support the findings of this study are available from the corresponding author upon reasonable request.
